# FOXO1 mitigates the SMAD3/FOXL2^C134W^ transcriptomic effect in a model of human adult granulosa cell tumor

**DOI:** 10.1186/s12967-021-02754-0

**Published:** 2021-02-27

**Authors:** Christian Secchi, Paola Benaglio, Francesca Mulas, Martina Belli, Dwayne Stupack, Shunichi Shimasaki

**Affiliations:** 1 Department of Obstetrics, Gynecology, and Reproductive Sciences, University of California, San Diego School of Medicine, 9500 Gilman Drive, La Jolla, CA 92093 USA; 2 Department of Pediatrics, University of California San Diego, School of Medicine, La Jolla, CA USA

**Keywords:** RNA-seq, FOXL2, SMAD3, FOXO1, Adult granulosa cell tumor, HGrC1

## Abstract

**Background:**

Adult granulosa cell tumor (aGCT) is a rare type of stromal cell malignant cancer of the ovary characterized by elevated estrogen levels. aGCTs ubiquitously harbor a somatic mutation in *FOXL2* gene, Cys134Trp (c.402C < G); however, the general molecular effect of this mutation and its putative pathogenic role in aGCT tumorigenesis is not completely understood. We previously studied the role of FOXL2^C134W^, its partner SMAD3 and its antagonist FOXO1 in cellular models of aGCT.

**Methods:**

In this work, seeking more comprehensive profiling of FOXL2^C134W^ transcriptomic effects, we performed an RNA-seq analysis comparing the effect of FOXL2^WT^/SMAD3 and FOXL2^C134W^/SMAD3 overexpression in an established human GC line (HGrC1), which is not luteinized, and bears normal alleles of *FOXL2*.

**Results:**

Our data shows that FOXL2^C134W^/SMAD3 overexpression alters the expression of 717 genes. These genes include known and novel FOXL2 targets (*TGFB2*, *SMARCA4*, *HSPG2*, *MKI67*, *NFKBIA*) and are enriched for neoplastic pathways (Proteoglycans in Cancer, Chromatin remodeling, Apoptosis, Tissue Morphogenesis, Tyrosine Kinase Receptors). We additionally expressed the FOXL2 antagonistic Forkhead protein, FOXO1. Surprisingly, overexpression of FOXO1 mitigated 40% of the altered genome-wide effects specifically related to FOXL2^C134W^, suggesting it can be a new target for aGCT treatment.

**Conclusions:**

Our transcriptomic data provide novel insights into potential genes (FOXO1 regulated) that could be used as biomarkers of efficacy in aGCT patients.

## Background

Granulosa cell tumors (GCTs) are a rare form of stromal cell malignant cancer accounting for one in twenty cases of ovarian cancer. GCTs can occur as either adult or juvenile subtypes [1]. Commonly the adult form of GCTs is diagnosed at an early stage and can be treated surgically. However, these tumors often recur, and are predominantly fatal in case of recurrence [2–4]. Current research is focused on discovering new molecular markers to predict the progression of aGCTs. Established cancer markers have no prognostic significance for this type of tumor. Indeed, while many types of tumors show prognostic induction of oncogenes or inactivation of tumor suppressor genes, aGCTs reveal a modestly impacted genome. The immunohistochemical evaluation of different oncogenes and tumor suppressors (*e.g.,* MYC, CDKN1A, ERBB2 and TP53) failed to detect a correlation with patient outcome [5, 6].

More than 400 tumors from aGCT patients of diverse ethnicities [7] showed one single mutation in the Forkhead box L2 transcription factor gene FOXL2: Cys134Trp (c.402C < G) [8], however its role in the onset of aGCTs is still matter of investigation [9–14]. The mutation is not present in other cancers. FOXL2 is expressed in the ovarian granulosa cells (GCs) and plays a key role during the development of female reproduction system and in its maintenance [15–17]. Potential FOXL2 targets revealed by ChIP and RT-PCR implicated its involvement in follicular and craniofacial development [18–20]. It has been also shown that FOXL2 affects collagen synthesis [21, 22]. Nicol et al*.* carried out the first ChIP-seq on mouse FOXL2^WT^ in order to find its direct target [23]. In 2011, Benayoun et al. demonstrated that FOXL2 plays a key role in the homeostasis of GCs and its failure leads to ovarian aging and tumorigenesis [24]. This mutation hyperactivates aromatase and downregulates GC apoptosis pathways [10]. Whole transcriptome analysis of 10 aGCT patient samples showed that the FOXL2^C134W^ acts as a hypomorphic mutant on the normally FOXL2^WT^ activated genes [25]. Another transcriptomic study on several stage 1 and stage 3 aGCT samples identified 24 genes whose expression significantly fluctuated between stages [26]. More recently, whole genome sequencing of a large aGCTs cohort indicated *TP53* and *DICER1* as potential drivers in these tumors and found a higher mutational burden in recurrent tumors, as compared to primary AGCTs [27]. However, these molecular events are not frequent as FOXL2^C134W^. Recent publications investigated the FOXL2^C134W^ pathogenicity from a transcriptional perspective. Notably, Carles et al. developed an inducible FOXL2^C134W^ stable luteinized cell line (SVOG3e) [28] which demonstrated that FOXL2^C134W^ mutation may precisely alter DNA binding specificity. The study revealed low numbers of target genes and the authors noted the SVOG3e cell line may not respond appropriately to FOXL2^C134W^. This may be possibly due to the absence of the needed partner SMAD3. Indeed, it is well known that SMAD3, a central element of the TGFβ pathway, is essential for the FOXL2^C134W^ activity [11, 29–36]. In line with this, a recent study by Weis-Banke et al. [37], showed that FOXL2^C134W^ and the TGFβ pathway are together two important potential therapeutic targets. Indeed, using FOXL2^C134W^ overexpression and silencing molecular approaches in a non-luteinized GC cell line (HGrC1) treated with TGFβ, they showed that SMAD4 is also an important FOXL2^C134W^ partner and the TGFβ/FOXL2^C134W^ molecular events trigger the expression of oncogenic, EMT, and stemness pathways in an aGCT model [37]. It is well accepted that TGFβ has an important role in tumor progression and involves SMAD mediators that control expression of hundreds of genes in different ways in diverse contexts and physiologies [38, 39] although additional investigations are needed to find its specific action(s) in aGCTs.

To further demonstrate the role of SMAD3 as a molecular partner of FOXL2^C134W^ in aGCT progression on a genome-wide level, we carried out RNA-seq analysis in HGrC1 overexpressing FOXL2^C134W^ together with SMAD3, compared to FOXL2^WT^/SMAD3 as a control. We further explored the FOXL2 antagonistic tumor-suppressive transcription factor FOXO1 [35] to study its influence on the SMAD3/FOXL2^C134W^ induced alterations. FOXO factors are considered as tumor suppressors for their parts in the initiation of apoptotic process and in the cell cycle arrest [40]. We report that FOXL2^C134W^ over-expression was associated with altered signaling pathways previously recognized by transcriptomic studies from GC in situ [13, 37], and additionally identified novel candidate FOXL2^C134W^ targets. Furthermore, we observed that FOXO1 strongly mitigated the overall FOXL2^C134W^ action with important implications for both aGCT onset and future therapeutic development.

## Materials and methods

### Plasmids and reagents

Expression plasmid encoding N-terminally Flag-tagged hFOXO1-3A (#13,508) was purchased from Addgene (Cambridge, MA, pCDNA3 backbone). FOXO1-3A has Ala residue substitutions at Thr-24, Ser-256 and Ser-319 and is thus constitutively active. The usage of this mutant was based on the fact that protein remains in the nucleus due to the inability of insulin/growth factor signaling to phosphorylate the mutated residues. Like FOXL2 wild type (FOXL2^WT^), the FOXL2^C134W^ mutant is also localized in the nucleus [9] and thus FOXO1-3A is the best construct for our transcriptomic study. N-terminally Flag-tagged human FOXL2wt (pCS2 + backbone) was kindly given by Dr. Louise Bilezikjian [33] and its mutant (FOXL2^C134W^) was made in our laboratory as previously reported [41]. The expression plasmids encoding human SMAD3 were kindly provided by Drs. Kohei Miyazono [42] and Louise Bilezikjian [33].

### HGrC1 cell culture

HGrC1 cells were kindly provided by Drs. T. Nakamura and A. Iwase, and their properties and derivation described earlier [43]. Notably, these cells bear two normal alleles of the FOXL2 gene [34]. Cells were cultured in a standard incubator at 37 °C in DMEM-F12 medium (catalog #12,400–024, Thermo Fisher Scientific, Waltham, MA) complemented with antibiotics (penicillin and streptomycin) and 10% FBS (#F6178, Sigma-Aldrich). HGrC1 cells at passage 14 were used for all the experiments in this study. HGrC1 cells seeded into 6-well tissue culture plates (1 × 10^6^ cells/well). Medium was replaced after 24 h with serum-free DMEM-F12 (complemented with antibiotics), and transfection was performed using the above described plasmids for 4 h with Lipofectamine 3000 reagents (catalog #L3000008, Thermo Fisher Scientific) following manufacturer’s procedures. After 4 h, the medium was replaced with new serum free DMEM-F12 and transfected cells were cultured at 37 °C for 24 h.

### RNA-seq data generation

After 4 h transfection and additional culture for 24 h in serum free DMEM-F12, HGrC1 cells (15 samples including 3 replicates for 5 conditions) were lysed using TRIzol reagent (catalog #15596026, Thermo Fisher Scientific) and RNA was extracted with Direct-zol RNA MiniPrep kit (#R2052, Zymo Research, Irvine, CA). RNA Quality control was performed by Agilent TapeStation (Additional file 1: Figure S1). RNA-seq libraries were prepared using the TrueSeq Stranded mRNA Library Prep (Illumina, catalog #20020595) and sequenced using an illumina HiSeq4000 Platform using 75-bp single-end reads, to an average of 22.9 ± 3.4 million reads per sample.

### Western Blot

For protein quantification, additional five samples that were cultured in parallel were lysed in lysis buffer (RIPA buffer, catalog #89901, Thermo Fisher Scientific), phosphatase inhibitor cocktail (#78420, Thermo Fisher Scientific) and protease inhibitor cocktail (#P8340, Sigma-Aldrich), and Pierce BCA protein assay kit was used for total protein quantification (#23227, Thermo Fisher Scientific). NuPAGE LDS sample buffer 4X (#NP0007, Thermo Fisher Scientific) and β-mercaptoethanol (#6010, Calbiochem, Billerica, MA) were added to cell lysates, and samples were denaturized at 95 °C for 5 min. Proteins separation occurred on 8–12% SDS-PAGE gels and with subsequent transfer to nitrocellulose membranes. Membranes were then incubated for 1 h with blocking solution (BSA, #A30075-100, Research Products International, Mount Prospect, IL), and with primary antibodies overnight at 4 °C. Membranes were washed three times, then incubated for 1 h with secondary antibodies conjugated to infrared fluorescent dyes excitable at 800 nm (IRDye 800CW, Li-COR-USA) to visualize the desired antigens using an 800 nm laser scanner (Odyssey, Licor, USA). Quantification of protein levels was performed by IR fluorescence detection (Odyssey, Licor, USA) and analysis was performed with ImageJ.

### RNA-seq bioinformatics analyses

Differential expression analysis was performed with RNA-seq [44]. To quantify transcript abundance for each sample we used salmon (1.1.0) [45] with default parameters and using hg38 set as the reference genome for reads mapping. The data was subsequently imported in R using the tximport package [46] and processed with the DESeq2 package [47]. A total of 37,787 genes were analyzed. For Principal Component Analysis we normalized the raw transcript counts using *variance stabilized transformation* (*vst* function from DESeq2) and applied the *plotPCA* function from DESeq2, using the default 500 most variable genes to compute the principal components as well as using all expressed genes. Loadings for each gene and each PC were extracted from the “rotation” field using the *pca* function in R from the top 500 most variable genes and divided by the sum of loadings for all genes in each PC. All heatmaps were generated using vst expression values and the heatmap package in R, excluding genes from over-expressed vectors. Differential expression analysis was performed using DESeq2 comparing different pairs of conditions, and a threshold of Benjamini–Hochberg corrected p-value of 0.05 was chosen to define differentially expressed genes for each comparison.

### Analysis of publicly available FOXL2 ChIP-Seq data

To estimate the fraction of genes with FOXL2 binding sites we used two publicly available human FOXL2 ChIP-seq dataset: one from TGFβ-treated HGrC1 cells ectopically expressing FOXL2 or FOXL2^C134W^ [37] and the other from an immortalized granulosa cell line (SVOG3e) with inducible expression of V5-tagged FOXL2^C134W^ protein [28]. For the first one (ChIP1), BAM files from FOXL2^C134W^ and the negative empty vector control were obtained from the authors and peaks were called using MACS2 [48] callpeak with default setting and using q-value cutoff of peak detection of 0.01 (– q 0.01). For the second (ChIP2), we obtained the peak coordinates from GEO (GSE126171) from 4 replicates at 12hrs induction of V5-tagged FOXL2^C134W^ and merged peaks coordinates of the replicates. Peaks BED files from the above ChIP-Seq were intersected with a ± 5 kb window from the transcription start site (TSS) of human genes (genecode v35 and genecode v19 respectively for ChIP1 and ChIP2) to identify genes with FOXL2^C134W^ binding around their promoter region (*n* = 9,939 and *n* = 11,697, respectively). Enrichment for genes with FOXL2 binding was calculated using Fisher’s exact test in R, comparing DEGs versus non-DEGs.

To better characterize differential gene expression between FOXL2^C134W^ and FOXL2^WT^ conditions, we performed enrichment analysis for differences in binding between FOXL2^C134W^ and FOXL2^WT^. To identify differential binding sites, we downloaded triplicate ChIP-Seq data from TGFβ-treated HGrC1 cells ectopically expressing FOXL2^WT^, FOXL2^C134W^ or empty vectors from GEO (GSE138496) [37]. Sequencing reads were filtered and trimmed for quality using Trim Galore! (http://www.bioinformatics.babraham.ac.uk/projects/trim_galore) and subsequently aligned to the hg38 reference genome using bowtie2 [49]. Duplicate reads were removed from the alignment using picard MarkDuplicates (http://broadinstitute.github.io/picard/). A consensus set of peaks was generated using MACS2 with parameters as above, from combined FOXL2 and FOXL2^C134W^ BAM files and using the empty vector BAMs as background. A total of 46,235 peaks were identified and, for each peak, the number of mapped reads in each triplicate of FOXL2^WT^and FOXL2^C134W^ was quantified using featureCounts [50]. The obtained matrix of peak x sample was used to determine differential peaks between FOXL2^WT^and FOXL2^C134W^ using DESeq2. Peaks were intersected with a ± 10 kb window from TSSs of human genes (genecode v35). Enrichment for genes with differential FOXL2 binding was calculated using Fisher’s exact test in R.

### Gene ontology enrichment analyses

For Gene Ontology Enrichment (GO) analyses, we used Metascape [51] with default parameters. To reduce the confounding effect on gene expression derived from variable overexpression of the proteins from the plasmids across samples, we removed the terms that were associated with the expression of FOXL2 and/or SMAD3. In order to do so, we performed Gene Set Enrichment Analysis (GSEA) and identified gene ontology terms significantly associated with FOXL2 and/or SMAD3. In detail, the GSEA tool (http://www.broad.mit.edu/gsea) for continuous phenotypes was used to identify gene annotation categories correlated with the genes of interest*.* For each gene of interest (FOXL2 or SMAD3), GSEA was run with the expression of that gene across samples set as the “continuous phenotype”; significant gene annotation sets with activity coordinated to the select gene were identified with adjusted *p*-value < 0.25. The list of significant gene annotation sets is provided in Additional file 2: Table S1. These annotation terms were removed from the results obtained with Metascape, *i.e.* from each list of terms enriched for differentially expressed genes throughout this investigation.

### Real-time PCR validation

An independent transfection experiment was carried out for qPCR validation as described above. RNA was extracted with Direct-zol RNA MiniPrep kit (#R2052, Zymo Research, Irvine, CA) following manufacturer’s protocol. High-Capacity cDNA Reverse Transcription Kit (#4368814, Thermo Fisher Scientific) was used to reverse transcribe 1 μg RNA. mRNA expression was quantified by q-RT-PCR amplification of cDNA using SYBR Green PCR Master Mix (#4309155, Thermo Fisher Scientific) and a Bio-Rad CFX384 Real-Time PCR Detection System. Q-RT-PCR was performed with primer assays from Sigma (#KSPQ12012G) and Qiagen (#330001) targeting the genes *TGFB1*, *TGFB2*, *ZYX*, *NFKBIA*, *SDC4*, *COL4A2*, *BAZ2A*, *SMARCA4*, and *HSPG2*. Primer assay efficiencies were guaranteed by the manufacturer. Target gene expression was normalized on GAPDH and ACTIN expression.

## Results

### Transcriptomics analysis of the human granulosa HGrC1 cell in presence of FOXL2^C134W^

**Fig. 1:**
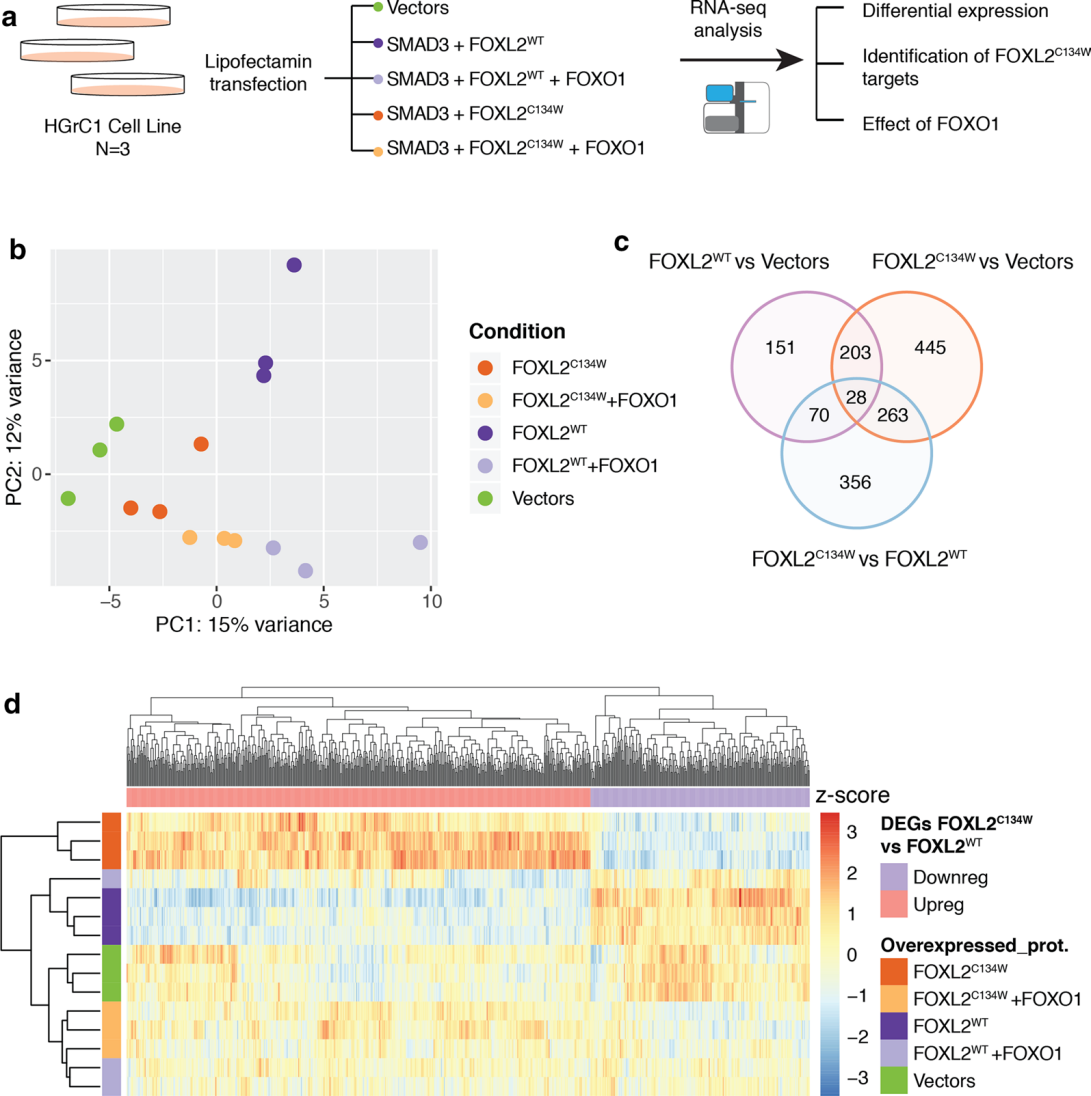
Transcriptome analysis of the human granulosa HGrC1 cell line under different transfection conditions. **a** Schematic summary of study design. FOXL2^WT^/FOXL2^C134W^ were in pCS2 + expression plasmid constructs; FOXO1^3A^ in pCDNA3 expression construct to minimize promoter competition. **b** Principal component 1 and 2 from all samples of the study, color-coded as indicated in the legend. Principal components were calculated on vst-normalized expression values from the top 500 most variable genes across samples. (SMAD3 was co-transfected with the other protein constructs per Methods but is omitted in the legend for brevity). **c** Venn diagram showing the number of differential genes (DESeq, q < 0.05) identified in 3 pairs of conditions: FOXL2^WT^ vs Vectors, FOXL2^C134W^ vs Vectors and FOXL2^C134W^ vs FOXL2^WT^. **d** Heatmap and hierarchical clustering of the 717 DEGs between FOXL2^C134W^ and FOXL2^WT^ in all 5 conditions. Expression values are z-score normalized across samples (columns)

To investigate the transcriptional changes associated with the SMAD3/FOXL2^C134W^ program, and its interaction with FOXO1, HGrC1 cells transiently expressing SMAD3 and FOXL2^WT^/FOXL2^C134W^, with or without FOXO1-3A (FOXO1 hereafter), were profiled using RNA sequencing (Fig. 1a). Additional file 1: Fig. S1 shows the efficiency of transfection and demonstrates balanced protein expression of FOXL2^WT^, FOXL2^C134W^ and FOXO1. Principal Component Analysis (PCA) was calculated on variance stabilized transformation (*vst*)-normalized expression values from the top 500 most variable genes across samples (Fig. 1b), as well as using all expressed 37,787 genes (Additional file 1: Figure S3). The variation between samples was largely explained by the de novo gene expression from overexpressing plasmids (Additional file 1: Fig. S3), with samples of the same conditions clustering closer together, indicating good reproducibility between replicates. In particular, *FOXO1*, *FOXL2*, and *SMAD3* were the top 3 genes contribution to the first 3 PCs (38% variance explained, Additional file 1: Figure S4). To identify genes selectively induced by FOXL2 or FOXL2^C134W^, we next assessed differential expression analysis between FOXL2^WT^ and controls, FOXL2^C134W^ and controls, and FOXL2^C134W^ and FOXL2^WT^, and identified a total of 452, 939 and 717 differential expressed genes (DEGs, q < 0.05, Additional file 2: Table S1) genes respectively, many of which were shared among the different conditions (Fig. 1c). Of note, FOXL2^C134W^ induced more changes with respect to the overexpression of the wild type protein, possibly indicating an enhanced binding and/or transcriptional capacity.

When performing hierarchical clustering of the 717 DEGs between FOXL2^C134W^ and FOXL2^WT^ in all 5 conditions (Fig. 1d), we observed different patterns of expression with respect to the control, with the most common pattern being genes upregulated in FOXL2^C134W^ with respect to FOXL2^WT^ and to the control, but not upregulated in FOXL2^WT^ respect to the controls. This suggests that a majority of the expression changes involved genes that were not canonical targets of FOXL2 wild type, but became target of gain-of-function FOXL2^C134W^, in line with a previous study by Weis-Banke et al. [37]. On the other hand, samples that overexpressed FOXO1 in addition to FOXL2^C134W^ or FOXL2^WT^ clustered closer to the controls than their counterpart without FOXO1, suggesting that FOXO1 attenuated the effects of overexpressing FOXL2^C134W^ or FOXL2^WT^. These two observations will be further investigated in the following analyses.

### Gene ontology analysis of DEGs of FOXL2^C134W^ vs FOXL2^WT^

**Fig. 2:**
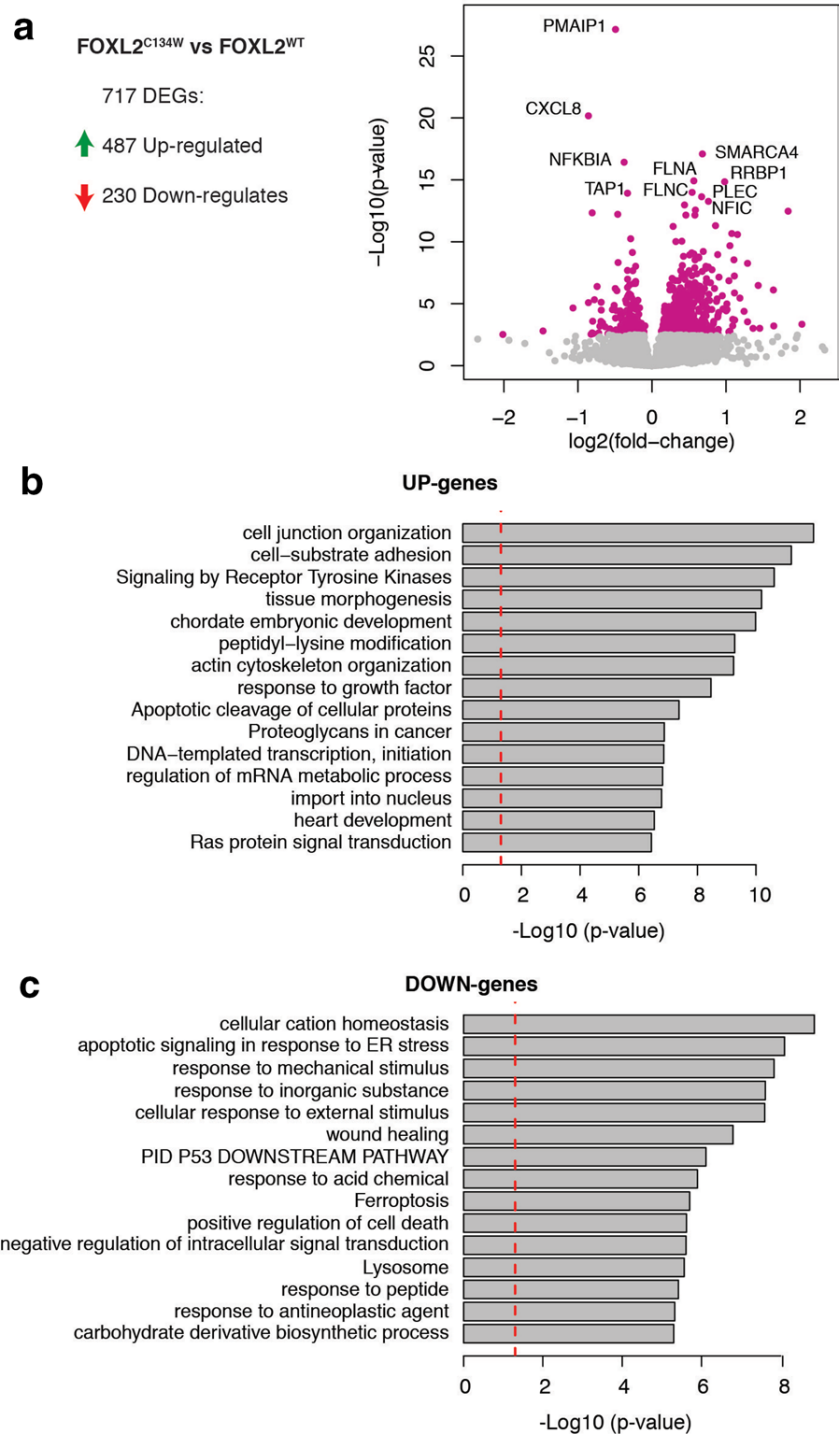
Gene Ontology enrichment analysis of differentially expressed genes (DEGs) between FOXL2^C134W^ and FOXL2^WT^. **a** Volcano plot showing in pink DEGs between FOXL2^C134W^ and FOXL2^WT^ (DESeq, q < 0.05). The genes FOXL2 and SMAD3 were omitted from the plot. **b**, **c** Gene ontology enrichment analysis using up-regulated genes in FOXL2^C134W^ transfected cells (**b**, n = 487), and down-regulated genes in FOXL2^C134W^ transfected cells (C, n = 230). The red line indicates p-value cutoff for p < 0.05. Gene ontology enrichment p-values were calculated using Metascape [51]. GO terms associated with expression of FOXL2 and/or SMAD3 were removed from the results (see Methods and Additional file 2: Tables S2–4)

To evaluate the functional significance of the genome-wide alterations in gene expression triggered by FOXL2^C134W^, we performed Gene Ontology (GO) analysis on DEGs, specifically focusing on the differences between FOXL2^C134W^ and FOXL2^WT^. The volcano plot (Fig. 2a) and heatmap analysis (Additional file 1: Fig. S5) revealed significantly upregulated (*e.g.* SMARCA4, RRBP1, FLNA) and downregulated genes (e.g. NFKBIA, TAP1, CXCL8). Among the upregulated and downregulated differentially expressed genes (DEGs), many are associated with the (ECM) extracellular matrix (HSPG2, COL4A2, COL5A1) or cellular interaction with it (FLNA, FLNC), chromatin remodeling (SMARCA4, EP400, BAZ2A), and PI3K/AKT and inflammation pathways (AMIGO2, XBP1).

Enrichment analysis [51] of pathways was performed on 487 upregulated (Fig. 2b) and 230 downregulated genes (Fig. 2c). The upregulated genes were highly enriched for gene annotation categories from oncogenic pathways, including Signaling by Receptor Tyrosine Kinases, and Proteoglycans in Cancer, Tissue Morphogenesis and response to growth factor. Interestingly, GO categories enriched for downregulated genes (Fig. 2c) included pathways often suppressed by oncogenes. These contained Wound Healing and the PID P53 Downstream pathways. Overall these data support the pathognomic role of FOXL2^C134W^/SMAD3 complex as a driver of specific and selective oncogenic molecular events (Genes and GO categories in Additional file 2: Table S2–4).

### Identification of direct FOXL2 targets among DEGs between FOXL2^C134W^ and FOXL2^WT^

**Fig. 3:**
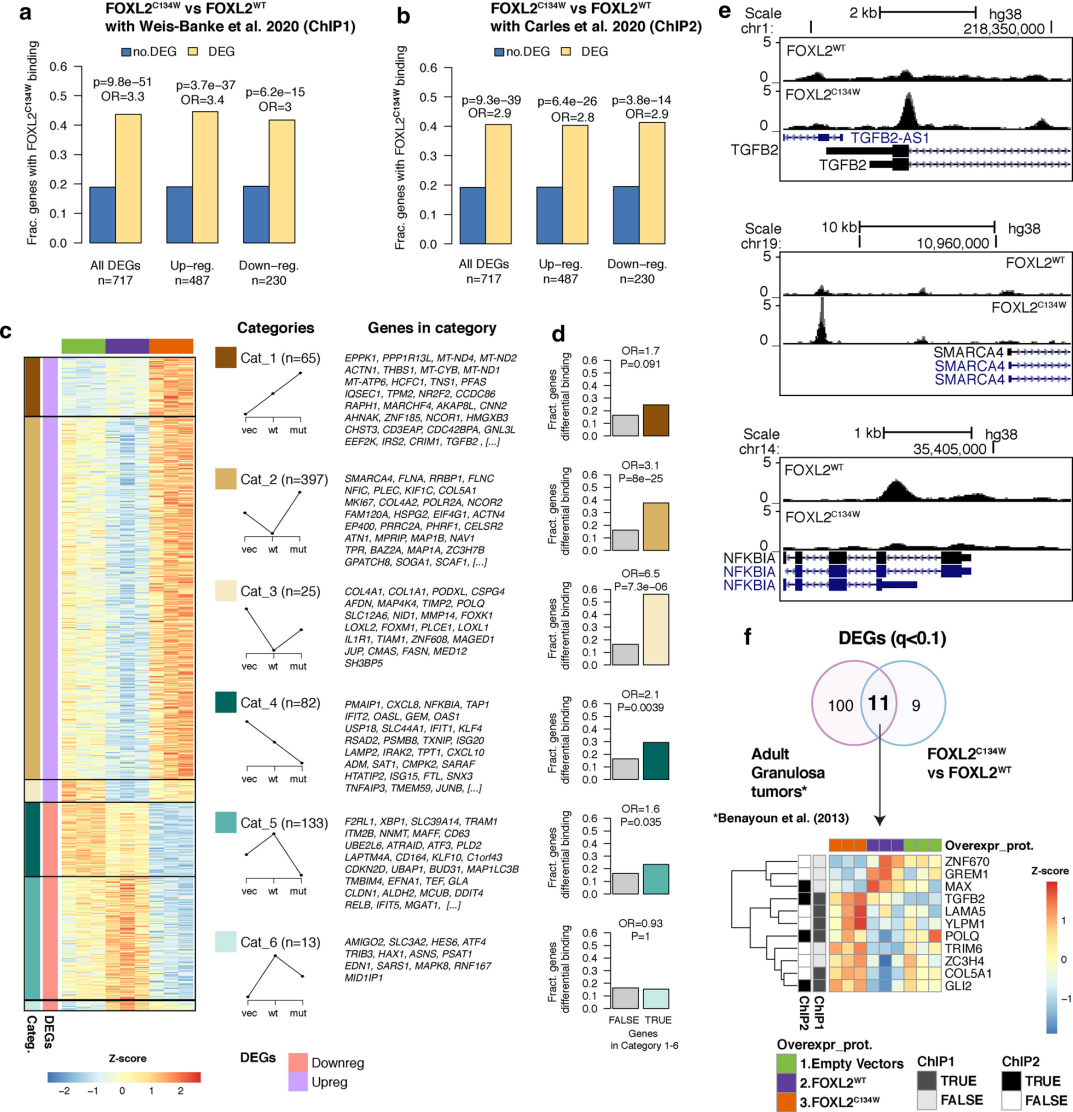
Identification of direct FOXL2 targets among DEGs between FOXL2^C134W^ and FOXL2^WT^. **a** Enrichment of FOXL2 binding in DEGs between FOXL2^C134W^ and FOXL2^WT^ with respect to non-DEGs using FOXL2^C134W^ ChIP-seq binding site in TGFβ-treated HGrC1 (ChIP1) [37] and another **b** using FOXL2^C134W^ ChIP-seq binding site in SVOG3e cell line (ChIP 2) [28]. p-values and odds ratios (OR) are from two-tailed Fisher’s exact test. **c** Heatmap of the 717 DEGs between FOXL2^C134W^ and FOXL2^WT^ grouped into 6 categories based on direction of expression in FOXL2^C134W^, FOXL2^WT^ and empty vector, as depicted in the diagrams. For each category, the top 30 DEGs (or less for smaller groups) are listed. **d** Enrichment of differential FOXL2^C134W^ vs FOXL2^WT^ binding (see Methods) at promoters of genes in each of the 6 DEG categories, color-coded as in (**c**). p-values and odds ratios (OR) are from two-tailed Fisher’s exact test. **e** Genome-browser screenshots of FOXL2^WT^ and FOXL2^C134W^ ChIP-Seq in TGFβ-treated HGrC1 [37], showing differential binding at the TGFB2 (top), SMARCA4 (middle) and NFKBIA (bottom) promoter regions. **f** Venn diagram (upper panel) showing the number of differential genes (q < 0.1) identified in FOXL2^C134W^ vs FOXL2^WT^ (20 DEGs) from the present study and from adult granulosa cell tumor patient samples vs control samples (111 DEGs) previously published [25]. The lower panel shows heatmap of the common identified 11 genes. For each gene, expression values were z-score normalized across samples. Genes are annotated for presence or absence of a FOXL2^C134W^ ChIP-seq binding site in TGFβ-treated HGrC1 (ChIP1) [37] and a FOXL2^C134W^ ChIP-seq binding site in a SVOG3e cell line (ChIP 2) [28]

To determine whether DEGs were direct targets of FOXL2^C134W^, we next annotated gene promoters using available FOXL2^C134W^ ChIP-seq from the recent studies by Weis-Banke et al. [37] (ChIP1) in TGFβ-treated HGrC1, and by Carles et al*.* [28], which included inducible expression of FOXL2^C134W^ in human SVOG3e cell line (ChIP2). A large fraction (44–40%) of DEGs between FOXL2^C134W^ and FOXL2^WT^ had a FOXL2^C134W^ binding site near their promoters (313 out of 717 using the HGrC1 ChIP-seq and 291 using the SVOG3e ChIP-Seq), which was a significantly higher proportion than in non-DEGs (Fisher’s exact test, Fig. 3a overall p = 9.8 × 10^–51^; upregulated p = 3.7 × 10^–37^; and downregulated p = 5.2 × 10^–15^; Fig. 3b overall p = 9.9 × 10^–39^; upregulated p = 6.4 × 10^–26^; and downregulated p = 3.8 × 10^–14^). The presence of these binding sites suggests that the changes in gene expression in our system are due in large extent to differences in FOXL2 binding.

To better characterize those targets differentially modified by FOXL2^C134W^, we grouped the 717 DEGs in six subsets (labeled as Cat_1 to Cat_6) of upregulated and downregulated genes according to different patterns of expression in FOXL2^C134W^ and FOXL2^WT^ conditions with respect to the vector, as depicted in Fig. 3c. For example, category 1 (Cat_1) included genes, such as *ACTN1* and *TGFB2,* that were upregulated in both mutant and wild-type conditions, while category 2 (Cat_2) included genes, such as *SMARCA4, HSPG2* and *BAZ2A*, that were upregulated only in FOXL2^C134W^.

To understand if these effects could be explained by differences in FOXL2 binding, rather over-expression artifacts, we analyzed the enrichment for differential ChIP-Seq peaks in each category (Fig. 3d). Differential peaks were calculated using DESeq2 between FOXL2^C134W^ and FOXL2^WT^ ChIP-Seq in TGFβ-treated HGrC1 from by Weis-Banke et al. [37]. Out of a total of 46,235 sites, we identified 12,515 differential peaks (q < 0.05), where 10,353 had stronger FOXL2^C134W^ signal (i.e. FOXL2^C134W^ showed a stronger binding in an existing FOXL2 site or bound to a completely new site), and only 2,162 had stronger FOXL2^WT^ signal. Examples of differential peaks at the *TGFB2*, *SMARCA4* and *NFKBIA* promoters are shown in Fig. 3e.

When comparing the fraction of genes with differential peaks in each category, we found that in the upregulated DEGs, Cat_2 and Cat_3, which had discordant effect with respect to the vector, had the highest enrichment (Fisher’s exact test, Fig. 3d, odds ratios = 3.1 and 6.5, and p = 8 × 10^–25^ and 7.3 × 10^–6^ respectively), while Cat_1, which had concordant effect with respect to the vector, was not significantly enriched for differential peaks (OR = 1.7, p = 0.091). These data suggest that the more dramatic changes in expression were more likely to be due to alteration of FOXL2 binding due to the gain-of-function mutation. For the down-regulated DEGs categories (Cat 4 to 6) we did not observe the same enrichment patterns, perhaps due to the smaller number of genes, or more complex mechanisms leading to gene repression.

Additionally, we performed GO enrichment analysis of the four largest categories of DEGs (Cat_1, Cat_2, Cat_4 and Cat_5, Additional file 1: Fig. S6) and found different enrichment for oncogenic regulating pathways such as *Cytoskeleto*n (Cat_1), *grow factor signaling* (Cat_2), *NF-kappaB and Tyrosine Kinase Signaling* (Cat_4), *Lysosome* (Cat_5). Additional file 1: Fig. S7 includes validation by qPCR for mRNA levels of several genes of these categories (TGFB1, TGFB2, ZYX, NFKBIA and SDC4) in an independent experiment showing that these gene followed the same pattern of our analysis.

To compare our findings with those from overexpression of FOXL2^C134W^ in the human SVOG3e cell line [28], we intersected our list of 939 DEGs of FOXL2^C134W^ vs. Vector with the corresponding published list of 471 DEGs, and identified 90 common genes (Additional file 1: Fig. S8). Among these genes, we found TGFB2, EGR1, NFKBIZ, SDC, and SDCBD which are homologues genes to the obtained ones in our previous analysis (Fig. 3c, d). Chip-seq data were also compared to the DEGs (Additional file 1: Fig. S7B). Gene Ontology analysis of these 90 genes revealed cancer-related regulatory pathways (Additional file 1: Fig. S8C).

To complete our comparative analysis with the most recent transcriptomic data, we also intersected our FOXL2^C134W^ and FOXL2^WT^ DEGs list (717 genes) to the those DEGs in Weis-Banke et al. (Additional file 1: Fig. S9). In addition, 29 common genes identified via Chip-seq data [37] were also compared to the DEGs (Additional file 1: Fig. S9A, B). Analysis of these genes showed an involvement reproduction and tumorigenesis-related GO pathways like cell-adhesion regulation and negative regulation of wound healing (Additional file 1: Fig. S9C). Finally, comparing our FOXL2^C134W^ and FOXL2^WT^ DEGs (717 genes) to transcriptomic data from aGTC patients published by Benayon et al. [25], we were able to categorize 11 genes (Fig. 3e), of which at least 3 (TGFB2, POLQ, GLI2) are *bona fide* FOXL2 targets [28, 37] (Fig. 3e). TGFB2 is the most consistent FOXL2^C134W^-modulated candidate and was present as target and upregulated genes in every analysis performed. These global results of our transcriptomic support the putative key role of FOXL2^C134W^ in aGCT tumorigenesis playing as regulator of oncogene (TGFB2, GLI2, EGR1) and as modulator of ECM genes (HSPG2, SDC4, COL5A1) and chromatin/nucleic acid remodeling (SMARCA4, BAZ2A).

### FOXO1 overexpression mitigates FOXL2^C134W^ gene expression profile.

**Fig. 4:**
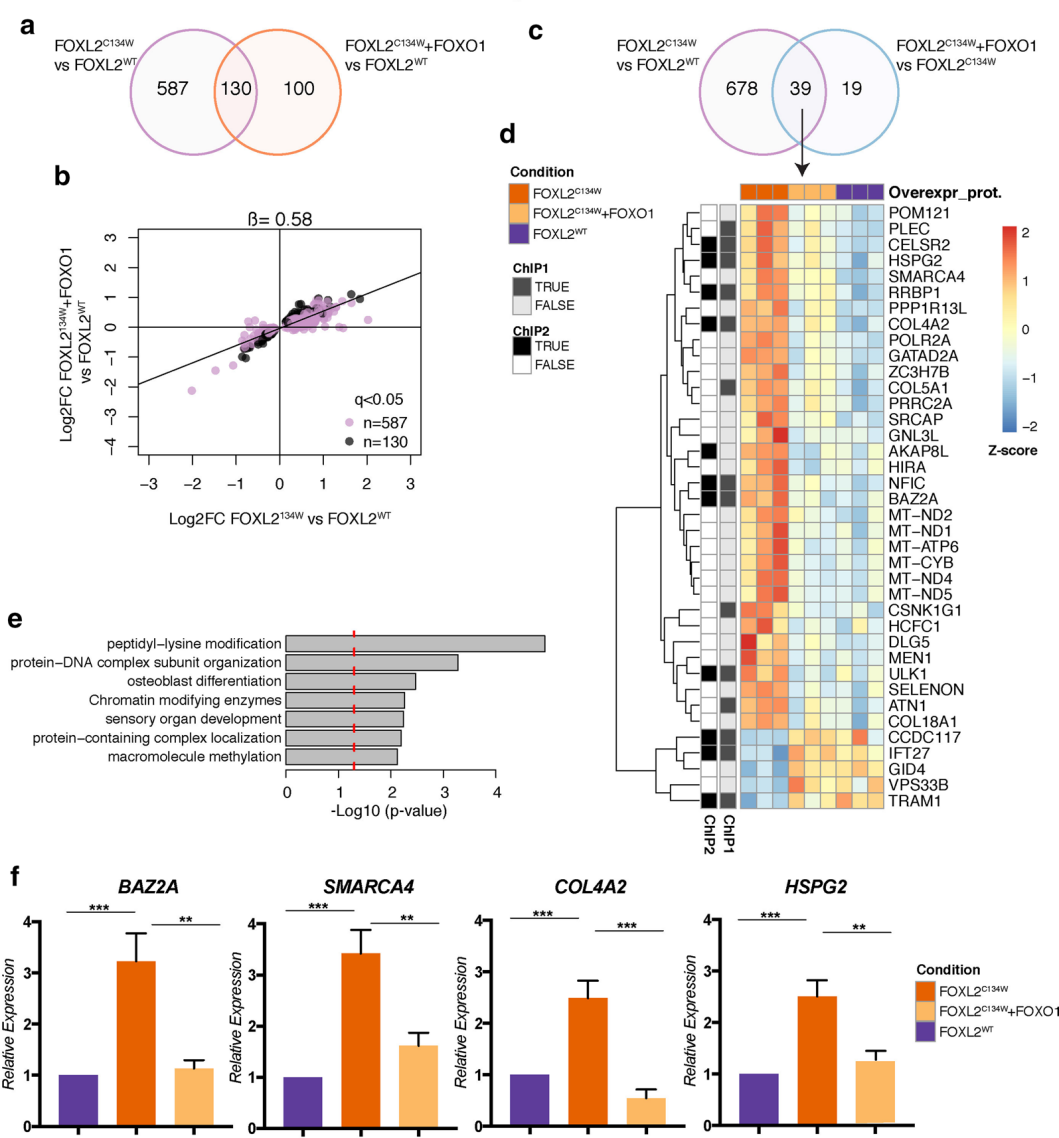
Effect of FOXO1 overexpression on FOXL2^C134W^ – modified genes. **a** Venn diagram showing the number of differential genes (DESeq, q < 0.05) identified in FOXL2^C134W^ vs FOXL2^WT^ and FOXL2^C134W^ + FOXO1 vs FOXL2^WT^. **b** Scatter plot of the effect sizes (log2-fold change) of DEGs between FOXL2^C134W^ and FOXL2^WT^, with (y-axis) and without (x-axis) the addition of FOXO1. Genes that were differentially expressed also when adding FOXO1^3A^ are shown in black (n = 130), while genes that were differentially expressed only without FOXO1^3A^ addition are shown in purple (n = 587). The slope (ß) of the linear relationship between the two effect sizes is shown on top of the plot. **c** Venn diagram showing the number of differential genes (DESeq, q < 0.05) identified in FOXL2^C134W^ vs FOXL2^WT^ and FOXL2^C134W^ + FOXO1 vs FOXL2^C134W^. **d** Heatmap with gene clustering of the 39 DEGs in both FOXL2^C134W^ vs FOXL2^WT^ and FOXL2^C134W^ + FOXO1 vs FOXL2^C134W^ comparisons. For each gene, expression values were z-score normalized across samples. Genes are annotated for presence or absence of a FOXL2^C134W^ ChIP-seq binding site in TGFβ-treated HGrC1 (ChIP1) [37] and a FOXL2^C134W^ ChIP-seq binding site in a SVOG3e cell line (ChIP 2) [28]. **e** Metascape GO analysis of the 39 DEGs in both FOXL2^C134W^ vs FOXL2^WT^ and FOXL2^C134W^ + FOXO1 vs FOXL2^C134W^ comparisons. The red line indicates p-value cutoff for p < 0.05. GO terms associated with expression of FOXL2 and/or SMAD3 were removed from the results (see Methods). **f** qPCR validation of the RNAseq expression data in FOXO1 modulated genes (BAZ2A, SMARCA4, COL4A2, and HSPG2). Graphs show fold change means ± s.e.m relative expression to FOXL^WT^ following normalization to the housekeeping gene. Data were analyzed using the two-tailed unpaired t-test (*p < 0.05, **p < 0.01, ***p < 0.001)

We next assessed the effect of FOXO1 overexpression in addition to FOXL2^C134W^ on gene expression profiles. Interestingly, the number of DEGs between FOXL2^C134W^ in the presence of FOXO1, and FOXL2^WT^ were much lower (230) relative to the number of DEGs between FOXL2^C134W^ and FOXL2^WT^ (717, of which 130 in common), suggesting that the transcriptional changes of FOXL2^C134W^ compared to FOXL2^WT^ are largely mitigated by the co-expression of FOXO1 (Fig. 4a). This notion is supported by comparing the overall effect size for DEG between FOXL2^C134W^ and FOXL2^WT^ (n = 717), with or without FOXO1 (Fig. 4b). While the log2FC are generally consistent across the two comparisons (FOXL2^C134W^ + FOXO1 vs FOXL2^WT^ and FOXL2^C134W^ vs FOXL2^WT^), the linear regression slope ß was 0.58 (less than 1), suggesting that up- or down-regulation of genes relative to the FOXL2^WT^ is attenuated by approximately 40% in presence of FOXO1, in our model system. A different estimation of this effect was measured by the absolute fold change of FOXL2^C134W^ + FOXO1 vs FOXL2^C134W^ at the 230 genes and resulted in an average of 15% modulation (± 19%, standard deviation). To further identify potential targets of FOXL2^C134W^ that are significantly modulated by FOXO1, we identified DEGs between FOXL2^C134W^ + FOXO1 and FOXL2^C134W^ and obtained 58 genes, having an average absolute fold change of 44%. Thirty-nine of these genes were in common with those between FOXL2^C134W^ and FOXL2^WT^ (Fig. 4c). These 39 genes (Fig. 4d), were downregulated in the FOXL2^C134W^ + FOXO1 vs FOXL2^C134W^ comparison, and vice versa. These genes were also compared against FOXL2 ChIP-seq from the recent studies by Weis-Banke et al. [37] and by Carles et al*.* [28]. Among these, 10 members were *bona fide* targets (including HSPG2, COL4A2 and BAZ2A). In particular, we identified the GO peptidyl-lysine modification pathway, implying that FOXO1 acts upon the chromatin remodeling triggered by FOXL2^C134W^. Moreover, other genes recognized in the ECM regulation were detected (HSPG2, COL4A2, COL5A1, COL18A1, DLG5). Independent qPCR validation of the BAZ2A, SMARCA4, COL4A2, and HSPG2 mRNA levels, modulated in presence of FOXO1, is depicted in Fig. 4f. These data build on our prior identification of FOXO1 as a modulator of FOXL2 and provide the first molecular basis for FOXO1 in aGCT reprogramming.

## Discussion

In contrast to the rarer juvenile Granulosa Cell tumors, the bulk of granulosa cell tumors are the adult type (aGCT), which occur peri to post-menopausally [52–54]. Patients characteristically present elevated estrogen levels and postmenopausal bleeding [3, 55]. Almost all aGCTs bear the unique somatic mutation FOXL2^C134W^ [56], which appears essential to the aGCT development [8, 9, 11, 25, 57–60]. However, its pathogenic role in the aGCTs has been investigated only recently and the FOXL2^C134W^ transcriptional action in aGCTs is still currently studied. These investigations are not trivial, as transcriptomic studies on rare tumors—like aGCTs—face difficulties in the procurement good quality RNA samples. Control samples from healthy human granulosa cells without any previous treatment (HGC or hormones) are similarly limited. Accordingly, only few transcriptomic explorations based on aGCTs samples are present in the literature [25, 26, 28, 37]. Carles et al*.* developed an inducible FOXL2^C134W^ stable luteinized cell line (SVOG3e) [28] showing FOXL2^C134W^ can precisely modify DNA binding specificity. More recently Weis-Banke et al. [37] characterized the genome-wide binding of overexpressed FOXL2^C134W^ and transcriptomic consequences, in presence of TGFβ on the same non-luteinized GC cell line (HGrC1) used in our study. They showed that mutant FOXL2 has an important partner not only in SMAD3, but also in SMAD4. This corroborates our previous hypothesis that the partnership between the FOXL2^C134W^ and TGFβ-pathway SMADs mediators is essential and specifically orchestrates the aGCT gene expression hallmarks [34, 35].

Here, we carried out a novel and complementary transcriptomic investigation overexpressing FOXL2^C134W^ in HGrC1 with its essential partner, SMAD3 [11, 29–36] to better understand its regulation and also avoiding other inevitable pleiotropic actions of the TGFβ stimulation (Fig. 1, Additional file 1: Fig. S1–4). Gene Ontology (GO) analysis on the 717 DEGs (FOXL2^wt^ vs. FOXL2^C134W^) revealed a clear engagement of pathways and genes related to tumorigenesis: Tissue Morphogenesis and Proliferation (MKI67, MYH9, FLNA), Regulation of the Extracellular Matrix (ECM) and Proteoglycans in Cancer (HSPG2, FLNC, COL4A2, COL5A1), Chromatin Remodeling with the Peptidyl-Lysine modification (SMARCA4, EP400, BAZ2A), downregulation of apoptotic pathway (ATF3, ATF4, CDKN2D, CXCL8), and PI3K/AKT pathway (AMIGO2, XBP1) (Fig. 2 and Additional file 1: Fig S5). Although MKI67, SMARCA4 and NF-kappaB pathway components have been described in aGCTs [5, 61–63] they have not previously been associated with FOXL2^C134W^ or implicated as potential direct targets.

Importantly, ~ 40% of the 717 DEGs had a FOXL2^C134W^ binding site at their promoters: 313 were identified using the TGFβ treated-HGrC1 ChIP-seq [37] (annotated ChIP1) and 291 using the SVOG3e ChIP-Seq [28] (ChIP2). This suggests that the variations in gene expression in our system were directly related to difference in FOXL2 binding (Fig. 3a, b). We considered to be important the comparison with FOXL2 ChIPseq analysis from Weis-Banke et al. (ChIP1) since it was validated with the hybrid DNA–binding sites in RNA extracted from 4 aGCT patients and, therefore, can be easily referred to as the best *bona fide* enrichment for FOXL2 for the aGCT investigation in the current literature.

Furthermore, we compared the binding sites of FOXL2^C134W^ and FOXL2^WT^ from Weis-Banke et al. [37] and identified 12,515 sites differentially bound (q < 0.05) by the two proteins: 10,353 had stronger FOXL2^C134W^ signal and only 2,162 had stronger FOXL2^WT^ signal, confirming strong gain-of-function of the mutated transcription factor. These differential sites were significantly associated with gene expression changes in our HGrC1 model overexpressing SMAD3, such as the striking examples of *TGFB2* and *SMARCA4* (Fig. 3e, overexpressed and with stronger FOXL2^C134W^ binding), and *NFKBIA* (Fig. 3e, downregulated and with stronger FOXL2^WT^ binding). We also found that these differential sites were more highly enriched at promoters of DEGs that were only regulated in FOXL2-^C134W^ background (i.e. were not upregulated in the overexpressed FOXL2^WT^), suggesting that these expression changes were more likely to be due to off target binding of the mutated FOXL2 than binding to canonical sites.

Interestingly, 11 genes–of which 3 (TGFB2, GLI2, POLQ) are direct FOXL2 targets according previous ChIP-seq studies—were observed to be common between the FOXL2^C134W^ and FOXL2^WT^ DEGs (717 genes) and the transcriptomic data from aGTC patients published by Benayon et al*.* [25] (Fig. 3f). 90 genes have been identified in common between our study and that of Carles et al*.* [28] (Additional file 1: Fig. S8, FOXL2^C134W^ vs Vectors condition). 29 common genes were also identified in common with the Weis-Banke et al. [37] (Additional file 1: Fig. S9, FOXL2^C134W^ vs FOXL2^WT^). It was remarkable that the TGBF2 gene emerges as the strongest FOXL2^C134W^ modulated candidate since was present as target and upregulated genes in all the analysis carried out. This is consistent with the molecular relationship between FOXL2 and TGFβ family [7, 41, 64]. Indeed, FOXL2^C134W^ can trigger neoplastic events in granulosa cells by altering the TGFβ pathway [7]. Importantly, this finding implies that FOXL2 mutant may drive the steady induction of its key molecular SMAD partners by via a direct TGFB2 positive feedback loop. aGCT therapeutic approaches based on the inhibition of the TGFβ family member Activin showed limited antitumoral activity. However, potentially clinically meaningful dose-related metabolic effects, including treatment of cancer cachexia, were observed that support further exploration of activin A inhibitors [65, 66]. Treatments based on the Activin inhibition and other novel TGFβ pathway targeting approaches–as strongly advocated by Weis-Banke et al. [37] and currently optimized for other indication [67–69]—will be necessary to better determine their clinical meaning in aGCT [65, 66]. Interestingly, the chromatin remodeler SMARCA4 (BSGR1)–already recognized as an important player in cellular tumorigenesis [70, 71] and expressed in all aGCT samples [62]–has been directly associated with TGFβ pathway [72] supporting the assumption of FOXL2^C134W^ as main pathognomic orchestrator of aGCT. Our overall data are a novel contribution in deciphering how FOXL2^C134W^ coordinates aGCT tumorigenesis.

An important result in our study came from the transcriptomic assessment of the onco-suppressor FOXO1 effect in our system. FOXO1 is the most abundantly expressed member of FOXO gene family, and regulates vital cellular processes including the cell cycle, apoptosis, energy homeostasis and ROS catabolism [73]. We previously studied the functional interaction between FOXO1, FOXL2^WT^ and FOXL2^C134W^ and it has been revealed the role of FOXO1 in the inhibition of CYP19 expression induced by the complex FOXL2^C134W^/SMAD3 [35]. Interestingly, FOXO1 had no influence on FOXL2^WT^ action, indicating that its inhibitory action targets selectively on FOXL2^C134W^-induced CYP19 expression in the presence of SMAD3 [34, 35]. The FOXO1 competition with FOXL2^C134W^ in binding SMAD3 was the suggested hypothetic underlying molecular mechanism since literature has showed that FOXO1 can partner with SMAD3 in different biological occurrences [74–77] and can sequestrate it from FOXL2^C134W^ [35, 78]. Insulin signaling through the PI3K-AKT pathway is the main modulator of FOXO1 [79]. FOXO1 phosphorylation during PI3K-AKT signaling promotes its translocation from the nucleus to cytoplasm and its consequent inactivation and proteasomal degradation [80]. The activation of PI3K-AKT signaling is common across many tumor types [81, 82] and the inactivation of FOXO1 in response to PI3K-AKT represents a common mechanism by which neoplastic cells prevent apoptosis and physiological cell cycle arrest [83–85]. It is well known that AKT inhibits FOXO through direct phosphorylation [86, 87]. Notably, FOXO1 can be indirectly targeted via the action of AKT inhibitors [88–91].

Our data revealed that ectopic expression of FOXO1 specifically and strongly moderated the overall effect of FOXL2^C134W^ compared to FOXL2^WT^, directly repressing the number of DEGs between FOXL2^C134W^ + FOXO1 and FOXL2^WT^ (Fig. 4a, b, f). This parallels our previous findings where our data indicated the inhibitory action of FOXO1 to SMAD3/FOXL2^C134W^ activity [35]. We identified potential targets of FOXL2^C134W^ that are significantly modulated by FOXO1 (58 genes, of which 39 in common) in the DEGs between FOXL2^C134W^ + FOXO1 and FOXL2^C134W^ (Fig. 4c, d) confirming that FOXO1 attenuates DEGs genes that were upregulated/downregulated by FOXL2^C134W^. We also identified 10 targets genes annotated in both ChIP-seq analysis [28, 37] (Fig. 4d). GO analysis of these genes (Fig. 4e) revealed FOXO1 modified similar pathways (peptidyl-lysine modification, chromatin remodeling, and ECM regulation) as those triggered by FOXL2^C134W^.

## Conclusions

**Fig. 5:**
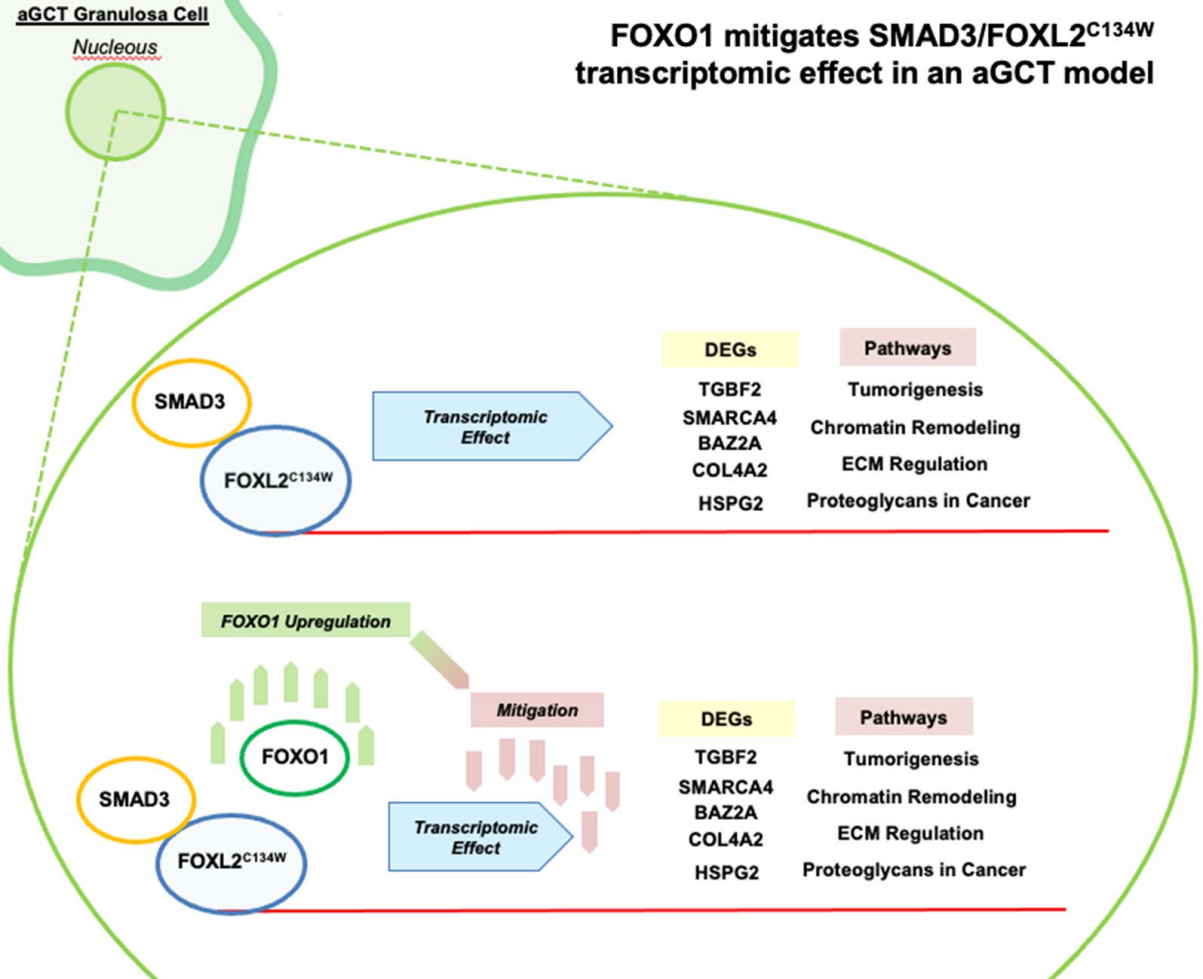
Graphic summary of the results and conclusions. According to our results in HGrC1 cells, the complex SMAD3/FOXL2^C134W^ drives a specific transcriptomic effect which involves genes (e.g. TGFB2, SMARCA4, BAZ2A, COL4A2 and HSPG2) related to neoplastic pathways such as Tumorigenesis, Chromatin remodeling, ECM regulation and Proteoglycans in Cancer, (top panel). FOXO1 upregulated expression obtained by Lipofection shows a significative mitigation of the SMAD3/FOXL2^C134W^ transcriptomic effect and suggests FOXO1 transcription factor as a new putative target for the aGCTs (bottom panel)

The comparative transcriptomic data presented in this investigation (Graphic summary depicted in Fig. 5) open new scenarios in understanding FOXL2^C134W^ mediate regulation of cell signaling, and illustrate: (i) that FOXL2^C134W^ can act to modulate important aGCT oncogenic pathways, including chromatin remodeling (SMARCA4, BAZ2A) and ECM regulation (HSPG2, COL4A2); (ii) the role of TGFB2, as upregulated effector and target gene in the aGCTs molecular occurrences; (iii) a potential molecular basis of emerging approaches targeting FOXO1 via AKT/PI3K pathway [92–94].

## Supplementary Information

**Additional file 1: Fig. S1**. FOXL2WT, FOXL2C134W and FOXO13A protein levels. After transfection with FOXL2WT, FOXL2C134W (both pCS2+ backbone) in presence/absence of FOXO13A (pCDNA3 backbone), cells were collected and lysate. (A) Immunoblot with anti-Flag tag antibody was performed to validate balanced FOXL2WT/C134W and FOXO1 protein expression. Anti β-Actin was used as loading control. (B) Quantification of protein levels was performed by IR fluorescence detection (Odyssey, Licor, USA) and analysis was performed with ImageJ. Fluorescence is expressed in arbitrary units.** Fig. S2**. RNA quality control assay. Representative diagrams of the quality control assay performed with the RNA ScreenTape® platform (Agilent Technology). Samples (N=3) were loaded and run. The analysis was performed with TapeStation Analysis Software (Agilent Technology). **Fig. S3. **PCA analysis. Scatter plot of principal components from the top 500 variable genes across the samples (A-B) or from all expressed genes (C-D), showing clustering based on transfection condition and expression of the transfected genes. Samples are color-coded based on transfection condition (1st column), FOXL2 expression (2nd column), FOXO1 expression (3rd column) and SMAD3 expression (4th column).** Fig. S4**. Top genes driving variability in the first 6 principal components. For PC 1 to 6, calculated on the top 500 most variable genes, we show the relative weight for each gene, ranked from highest to lowest. The top 8 genes contributing to each PC are indicated.** Fig. S5. **Top 50 regulated genes between FOXL2C134W and FOXL2WT ordered by p-value. (A) Heatmaps of the top 50 up-regulated genes and (B) the top 50 down-regulated genes between FOXL2C134W and FOXL2WT. The genes FOXL2 and SMAD3 were omitted form the plot. Genes are ordered by p-value (DESeq<0.05) and annotated for presence or absence of FOXL2C134W ChIP-seq binding site in TGFβ-treated HGrC1 (ChIP1) [37] and a FOXL2C134W ChIP-seq binding site in a SVOG3e cell line (ChIP 2) [28]. For each gene, expression values were z-score normalized across samples.** Fig. S6**. Gene Ontology enrichment analysis of the selected subset of differentially expressed genes (DEGs) between FOXL2C134W and FOXL2WT. Gene ontology enrichment analysis in the subsets of upregulated (Cat_1, n=60, Cat_2, n=397) and downregulated (Cat_4, n=82, Cat_5, n=133) genes in which the expression in FOXL2C134W conditions was in the same (Cat_1 and Cat_4) or opposite (Cat_2 and Cat_5) direction of FOXL2WT with respect to the vector. The red line indicates p-value cutoff for p<0.05. Gene ontology enrichment p-values were calculated using Metascape [51].** Fig. S7**. qPCR validation of the RNAseq expression data in TGFB1, TGFB2, ZYX, NFKBIA and SDC4 genes. Graphs show fold change means +/- s.e.m relative expression to FOXLWT following normalization to the housekeeping gene. Data were analyzed using the two-tailed unpaired t-test (*p<0.05, **p<0.01 and ***p<0.001).** Fig. S8**. Comparison with the DEGs FOXL2C134W vs Vectors from Carles et al. 2020. FOXL2C134W vs Vector DEGs were compared to previous published data at the same condition (Carles et al. 2020) [28]. A) Venn diagram showing the number of differential genes (DESeq, q<0.05) identified in FOXL2C134W vs Vectors (939 DEGs) from the present study and from Carles et al. 2020 (471 DEGs) [28]. B) Heatmaps of the top selected-50 of the total common 90 genes between FOXL2C134W and Vectors from our data and [28]. Genes are annotated for presence or absence of FOXL2C134W ChIP-seq binding site in TGFβ-treated HGrC1 (ChIP1) [37] and a FOXL2C134W ChIP-seq binding site in a SVOG3e cell line (ChIP 2) [28]. C) Metascape GO analysis of the 90 DEGs in both FOXL2C134W vs Vectors condition. The red line indicates p-value cutoff for p<0.05.** Fig. S9**. Comparison of the DEGs FOXL2C134W vs FOXL2WT from Weis-Banke S.E. et al. FOXL2C134W vs FOXL2WT DEGs were compared to previous published data at the same condition (Weis-Banke S.E. et al.) [37]. A) Venn diagram showing the number of differential genes (DESeq, q<0.05) identified in FOXL2C134W vs FOXL2WT (717 DEGs) from the present study and from Weis-Banke S.E. et al. [37] (537 DEGs). B) Heatmaps of the common 29 genes between FOXL2C134W and FOXL2WT from our data and [37]. Genes are annotated for presence or absence of a FOXL2C134W ChIP-seq binding site in [28, 37]. C) Metascape GO analysis of the 29 DEGs in both FOXL2C134W vs FOXL2WT condition. The red line indicates p-value cutoff for p<0.05.

**Additional file 2: Table S1**. List of the significant gene annotation sets. **Tables S2–S4**: Genes and GO Categories.

## Data Availability

Datasets generated in this study are available through Gene Expression Omnibus accession GSE166794.

## Abbreviations

aGCT: Adult-type granulosa cell tumor
DEGs: Differentially expressed genes
DMEM-F12: Dulbecco’s modified Eagle medium – F12
FOXL2: Forkhead box L2
FOXO1: Forkhead box O1
GC: Granulosa cell
GCT: Granulosa cell tumor
mRNA: Messenger RNA
PCR: Polymerase chain reaction
RT-PCR: Quantitative reverse transcription polymerase chain reaction
SBE: SMAD binding element
SEM: Standard error of the mean
