# Supplementary material for: FOXO1 mitigates the SMAD3/FOXL2C134W transcriptomic effect in a model of human adult granulosa cell tumor
Source: J Transl Med. 2021 Feb 27;19:90. doi: 10.1186/s12967-021-02754-0 (PMC7913442; doi:10.1186/s12967-021-02754-0)
Supplement: Supplementary file 1 — Additional file 1: Fig. S1. FOXL2WT, FOXL2C134W and FOXO13A protein levels. After transfection with FOXL2WT, FOXL2C134W (both pCS2+ backbone) in presence/absence of FOXO13A (pCDNA3 backbone), cells were collected and lysate. (A) Immunoblot with anti-Flag tag antibody was performed to validate balanced FOXL2WT/C134W and FOXO1 protein expression. Anti β-Actin was used as loading control. (B) Quantification of protein levels was performed by IR fluorescence detection (Odyssey, Licor, USA) and analysis was performed with ImageJ. Fluorescence is expressed in arbitrary units. Fig. S2. RNA quality control assay. Representative diagrams of the quality control assay performed with the RNA ScreenTape® platform (Agilent Technology). Samples (N=3) were loaded and run. The analysis was performed with TapeStation Analysis Software (Agilent Technology). Fig. S3. PCA analysis. Scatter plot of principal components from the top 500 variable genes across the samples (A-B) or from all expressed genes (C-D), showing clustering based on transfection condition and expression of the transfected genes. Samples are color-coded based on transfection condition (1st column), FOXL2 expression (2nd column), FOXO1 expression (3rd column) and SMAD3 expression (4th column). Fig. S4. Top genes driving variability in the first 6 principal components. For PC 1 to 6, calculated on the top 500 most variable genes, we show the relative weight for each gene, ranked from highest to lowest. The top 8 genes contributing to each PC are indicated. Fig. S5. Top 50 regulated genes between FOXL2C134W and FOXL2WT ordered by p-value. (A) Heatmaps of the top 50 up-regulated genes and (B) the top 50 down-regulated genes between FOXL2C134W and FOXL2WT. The genes FOXL2 and SMAD3 were omitted form the plot. Genes are ordered by p-value (DESeq<0.05) and annotated for presence or absence of FOXL2C134W ChIP-seq binding site in TGFβ-treated HGrC1 (ChIP1) [37] and a FOXL2C134W ChIP-seq binding site in a SVOG3e cell line (ChIP [file 12967_2021_2754_MOESM1_ESM.docx]

**Additional file 1: Figs.**

**
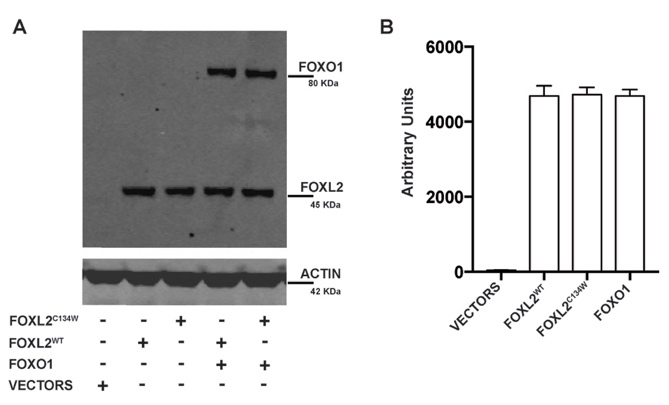
**

**Additional file 1: Fig. S1. FOXL2^WT^, FOXL2^C134W^ and FOXO1^3A^ protein levels.** After transfection with FOXL2^WT^, FOXL2^C134W^ (both pCS2+ backbone) in presence/absence of FOXO1^3A^ (pCDNA3 backbone), cells were collected and lysate. (A) Immunoblot with anti-Flag tag antibody was performed to validate balanced FOXL2^WT/C134W^ and FOXO1 protein expression. Anti β-Actin was used as loading control. (B) Quantification of protein levels was performed by IR fluorescence detection (Odyssey, Licor, USA) and analysis was performed with ImageJ. Fluorescence is expressed in arbitrary units.

**
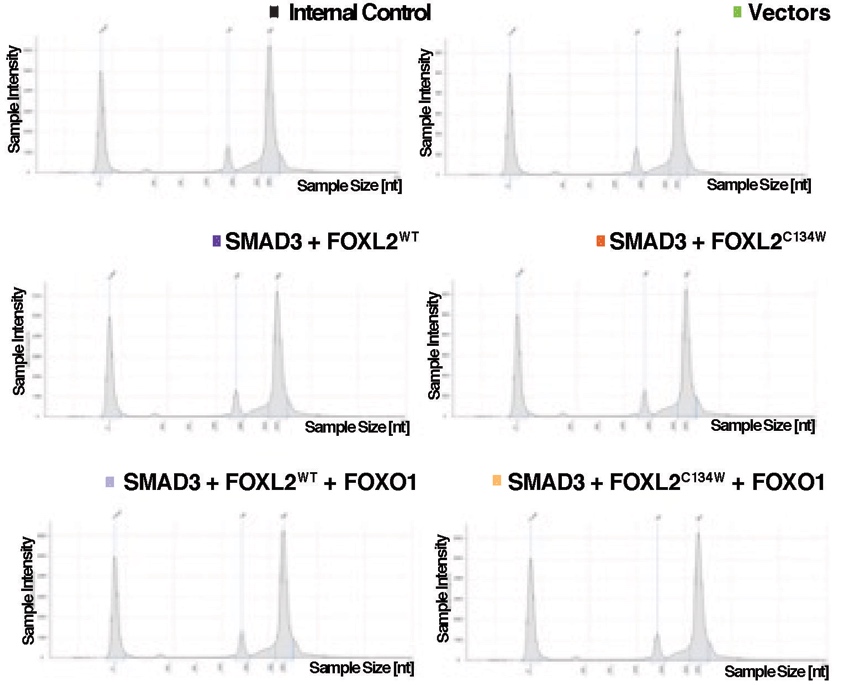
**

**Additional file 1: Fig. S2. RNA quality control assay.** Representative diagrams of the quality control assay performed with the RNA ScreenTape® platform (Agilent Technology). Samples (N=3) were loaded and run. The analysis was performed with TapeStation Analysis Software (Agilent Technology).


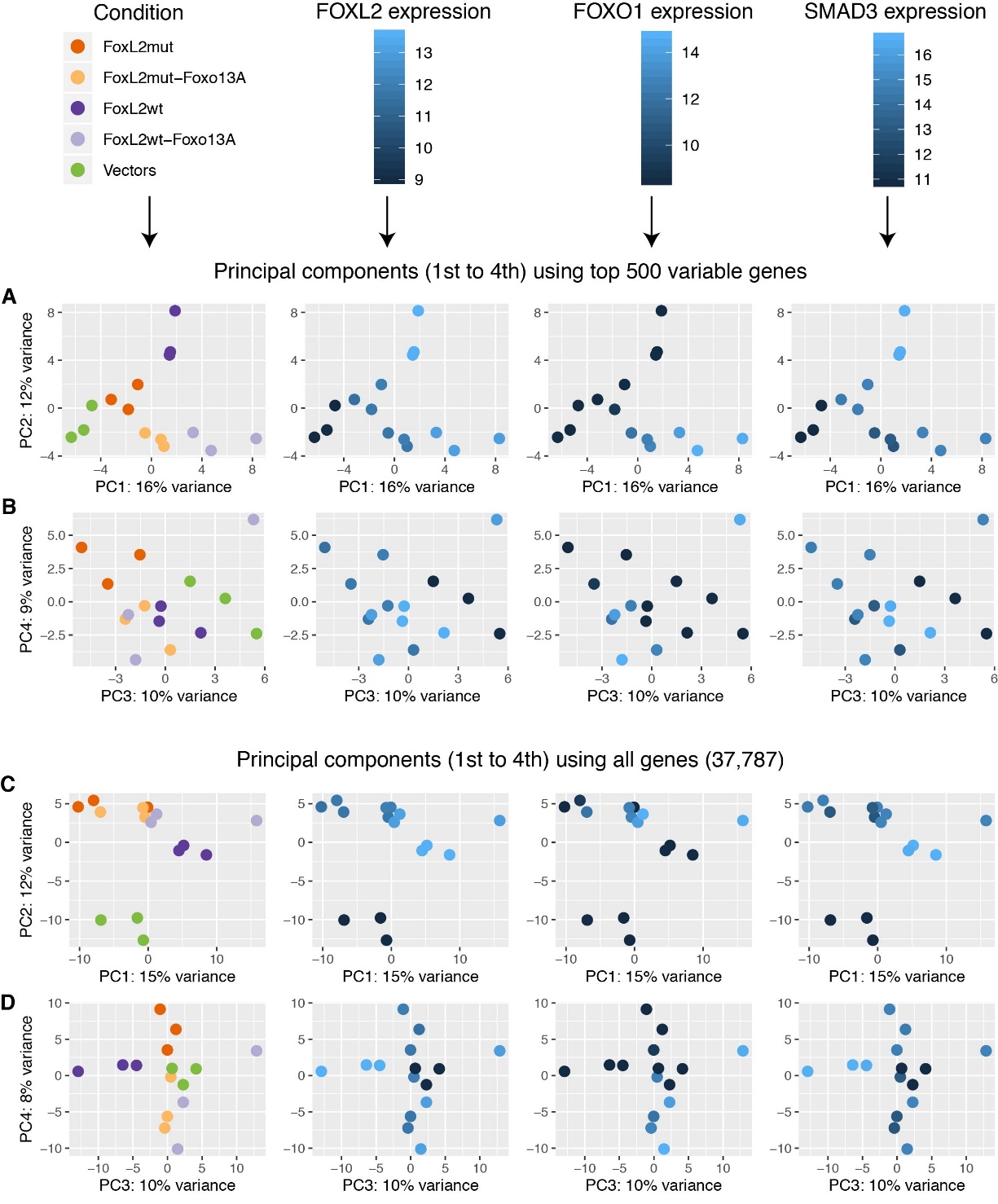


**Additional file 1: Fig. S3. PCA analysis.** Scatter plot of principal components from the top 500 variable genes across the samples (A-B) or from all expressed genes (C-D), showing clustering based on transfection condition and expression of the transfected genes. Samples are color-coded based on transfection condition (1^st^ column), *FOXL2* expression (2^nd^ column), *FOXO1* expression (3^rd^ column) and *SMAD3* expression (4^th^ column).

**
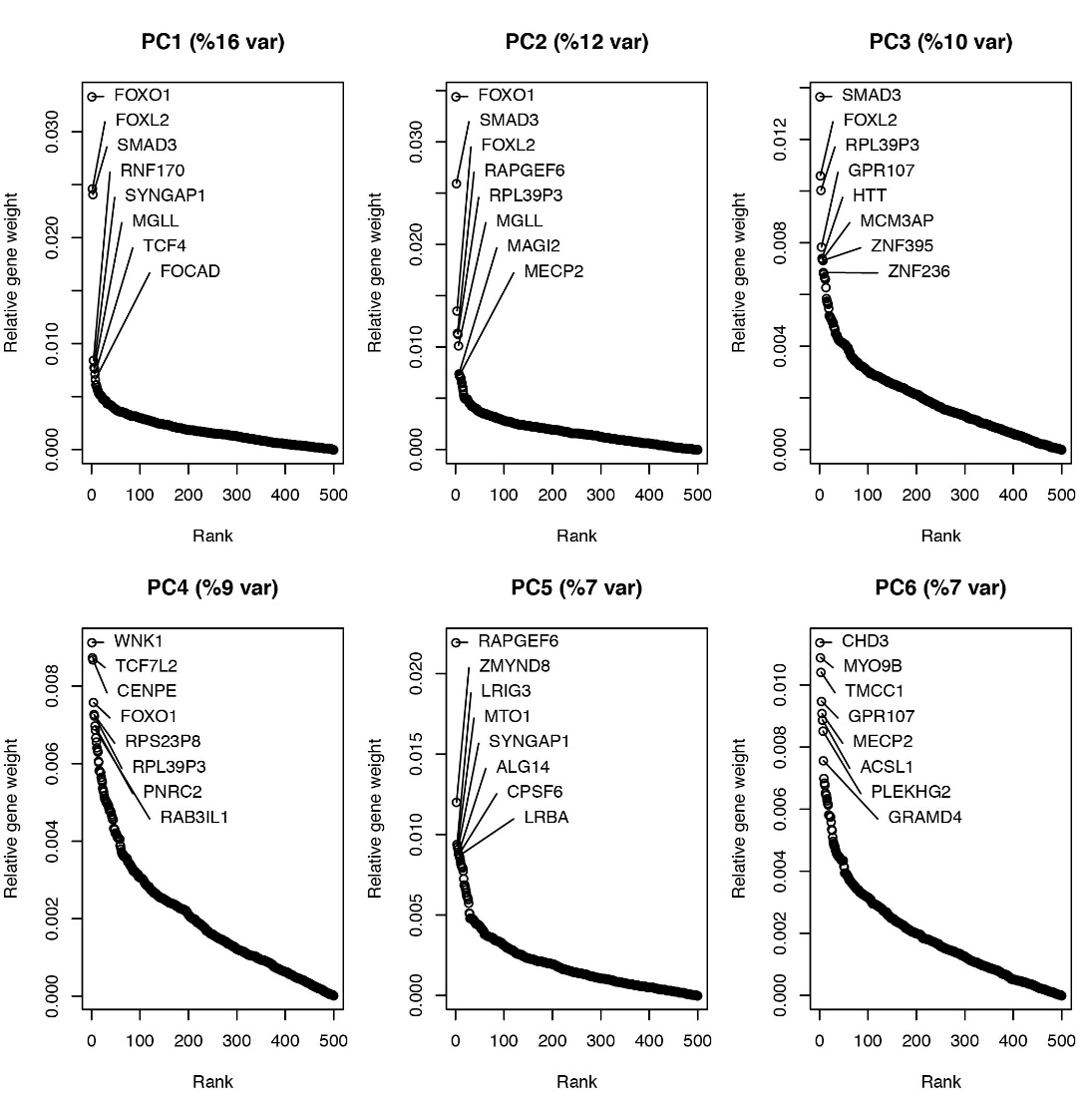
**

**Additional file 1: Fig. S4. Top genes driving variability in the first 6 principal components.** For PC 1 to 6, calculated on the top 500 most variable genes, we show the relative weight for each gene, ranked from highest to lowest. The top 8 genes contributing to each PC are indicated.

**
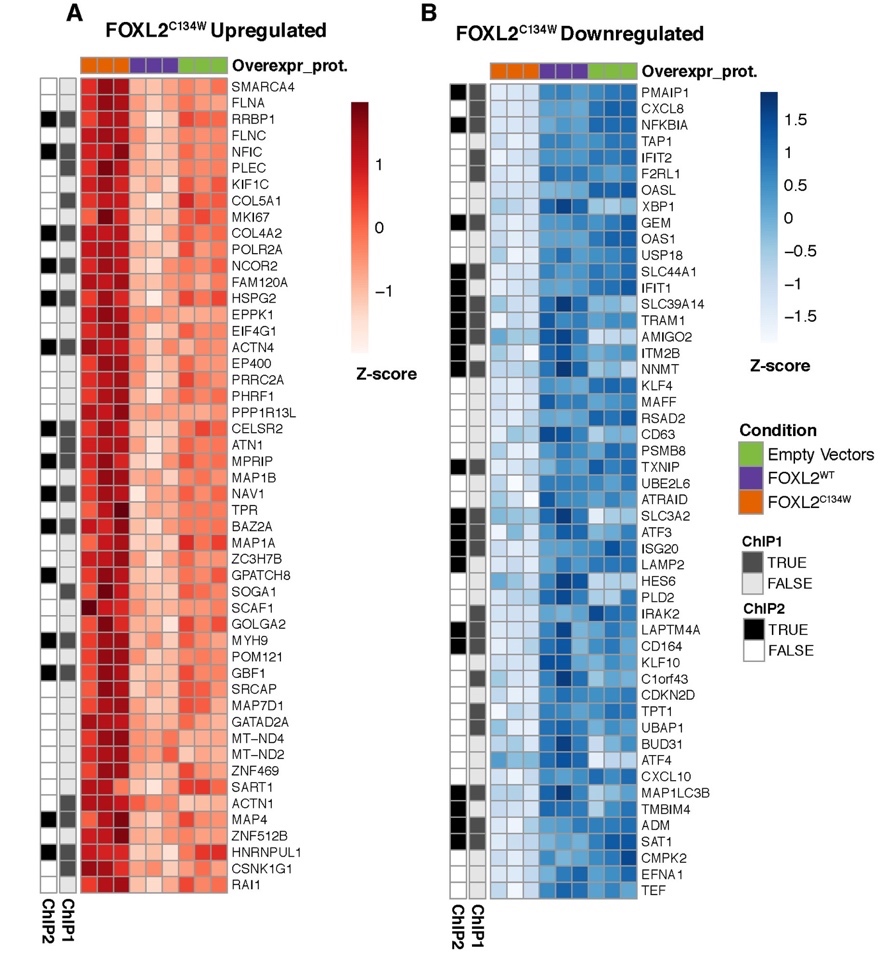
**

**Additional file 1: Fig. S5. Top 50 regulated genes between FOXL2^C134W^ and FOXL2^WT^ ordered by p-value.** (A) Heatmaps of the top 50 up-regulated genes and (B) the top 50 down-regulated genes between FOXL2^C134W^ and FOXL2^WT^. The genes FOXL2 and SMAD3 were omitted form the plot. Genes are ordered by p-value (DESeq<0.05) and annotated for presence or absence of FOXL2^C134W^ ChIP-seq binding site in TGFβ-treated HGrC1 (ChIP1) [37] and a FOXL2^C134W^ ChIP-seq binding site in a SVOG3e cell line (ChIP 2) [28]. For each gene, expression values were z-score normalized across samples.


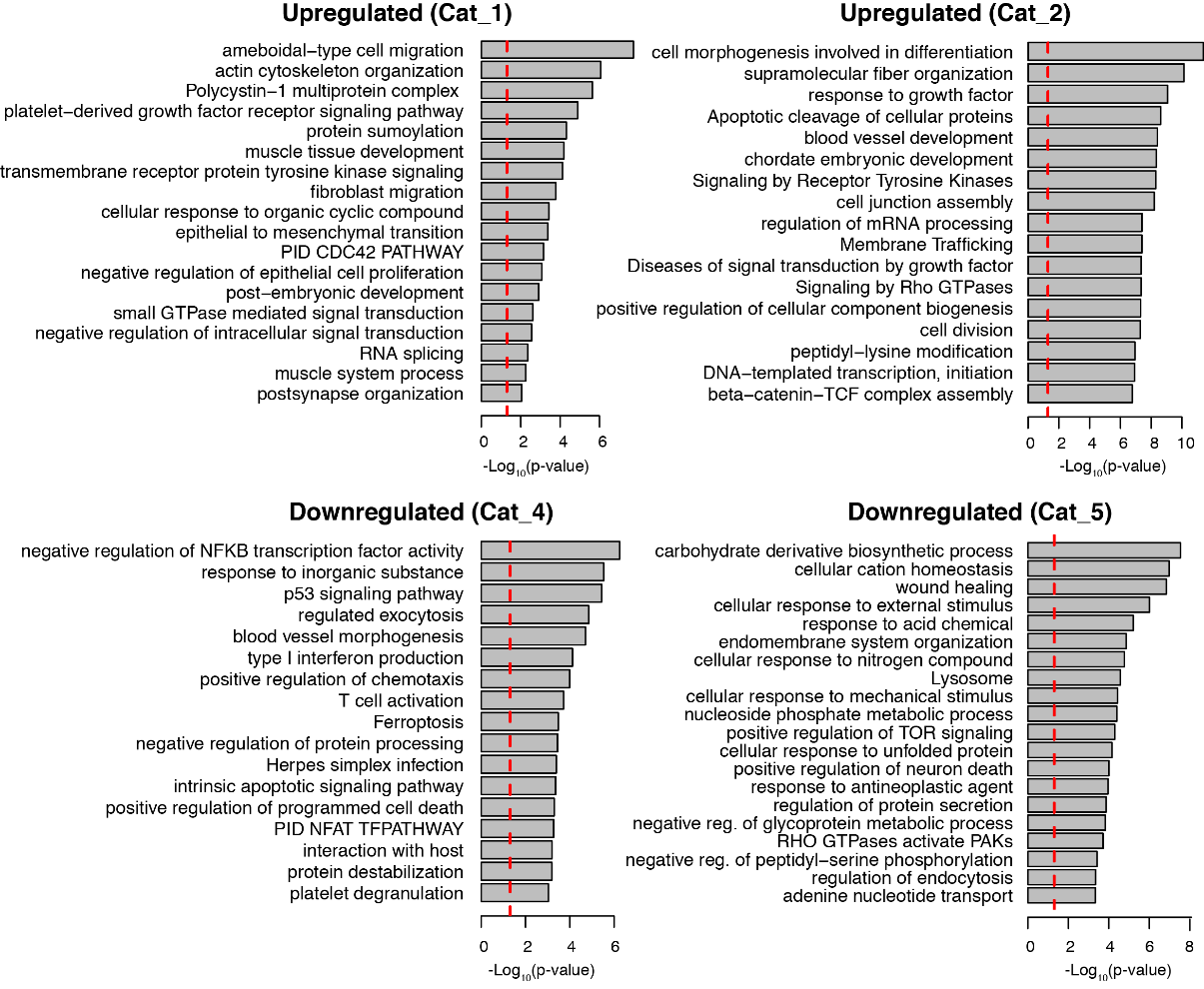


**Additional file 1: Fig. S6. Gene Ontology enrichment analysis of the selected subset of differentially expressed genes (DEGs) between FOXL2^C134W^ and FOXL2^WT^.** Gene ontology enrichment analysis in the subsets of upregulated (Cat_1, n=60, Cat_2, n=397) and downregulated (Cat_4, n=82, Cat_5, n=133) genes in which the expression in FOXL2^C134W^ conditions was in the same (Cat_1 and Cat_4) or opposite (Cat_2 and Cat_5) direction of FOXL2^WT^ with respect to the vector. The red line indicates p-value cutoff for p<0.05. Gene ontology enrichment p-values were calculated using Metascape [51].

**
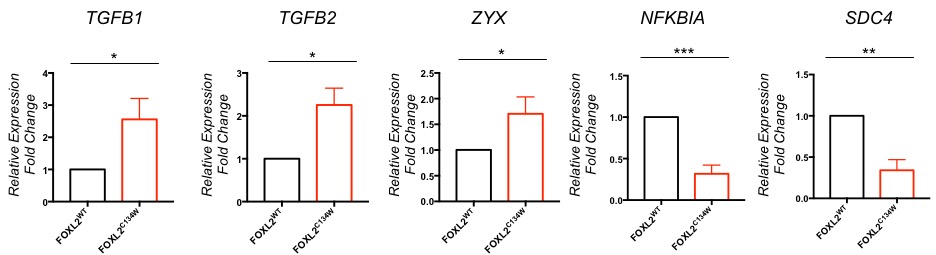
**

**Additional file 1: Fig. S7. qPCR validation of the RNAseq expression data in TGFB1, TGFB2, ZYX, NFKBIA and SDC4 genes.** Graphs show fold change means +/- s.e.m relative expression to FOXL^WT^ following normalization to the housekeeping gene. Data were analyzed using the two-tailed unpaired t-test (*p<0.05, **p<0.01 and ***p<0.001).

**
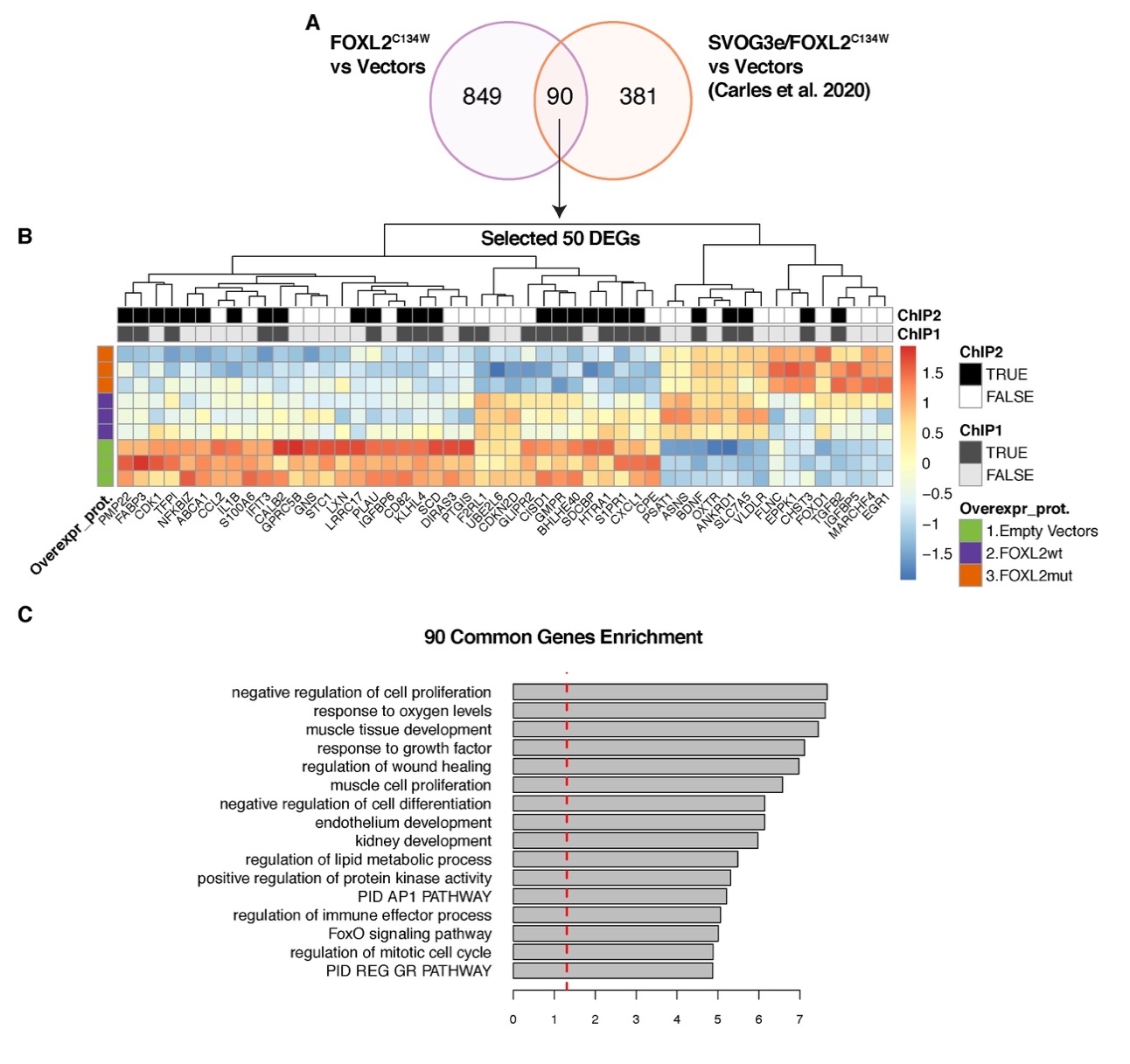
**

**Additional file 1: Fig. S8. Comparison with the DEGs FOXL2^C134W^ vs Vectors from Carles *et al.* 2020.** FOXL2^C134W^ vs Vector DEGs were compared to previous published data at the same condition (Carles *et al.* 2020) [28]. A) Venn diagram showing the number of differential genes (DESeq, q<0.05) identified in FOXL2^C134W^ vs Vectors (939 DEGs) from the present study and from Carles *et al.* 2020 (471 DEGs) [28]. B) Heatmaps of the top selected-50 of the total common 90 genes between FOXL2^C134W^ and Vectors from our data and [28]. Genes are annotated for presence or absence of FOXL2^C134W^ ChIP-seq binding site in TGFβ-treated HGrC1 (ChIP1) [37] and a FOXL2^C134W^ ChIP-seq binding site in a SVOG3e cell line (ChIP 2) [28]. C) Metascape GO analysis of the 90 DEGs in both FOXL2^C134W^ vs Vectors condition. The red line indicates p-value cutoff for p<0.05.

**
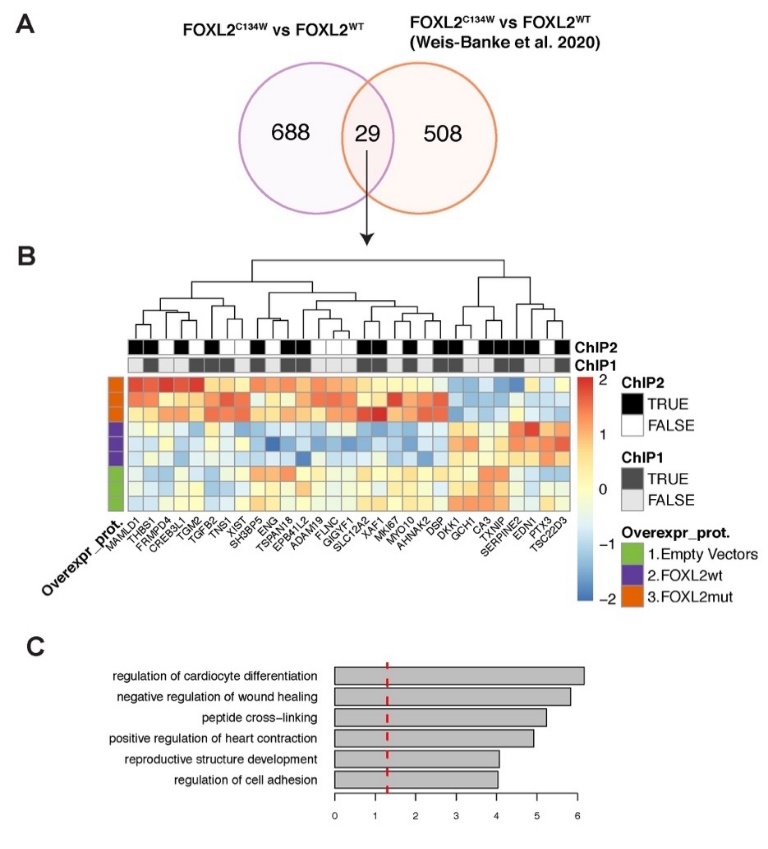
**

**Additional file 1: Fig. S9. Comparison of the DEGs FOXL2^C134W^ vs FOXL2^WT^** **from Weis-Banke S.E. *et al*.** FOXL2^C134W^ vs FOXL2^WT^ DEGs were compared to previous published data at the same condition (Weis-Banke S.E. *et al*.) [37]. A) Venn diagram showing the number of differential genes (DESeq, q<0.05) identified in FOXL2^C134W^ vs FOXL2^WT^ (717 DEGs) from the present study and from Weis-Banke S.E. *et al*. [37] (537 DEGs). B) Heatmaps of the common 29 genes between FOXL2^C134W^ and FOXL2WT from our data and [37]. Genes are annotated for presence or absence of a FOXL2^C134W^ ChIP-seq binding site in [28, 37]. C) Metascape GO analysis of the 29 DEGs in both FOXL2^C134W^ vs FOXL2^WT^ condition. The red line indicates p-value cutoff for p<0.05.
